# Comparative analysis of genetic risk scores for predicting biochemical recurrence in prostate cancer patients after radical prostatectomy

**DOI:** 10.1186/s12894-024-01524-6

**Published:** 2024-07-02

**Authors:** Ai-Ru Hsieh, Yi-Ling Luo, Bo-Ying Bao, Tzu-Chieh Chou

**Affiliations:** 1 Department of Statistics, Tamkang University, New Taipei City, 251301 Taiwan; 2 Department of Public Health, College of Public Health, China Medical University, Taichung, 40402 Taiwan; 3 School of Pharmacy, China Medical University, Taichung, 406040 Taiwan; 4 Department of Nursing, Asia University, Taichung, 41354 Taiwan; 5 Department of Health Risk Management, College of Public Health, China Medical University, Taichung, 40402 Taiwan

**Keywords:** Genetic risk score, Genome-Wide Association Study, Prostate cancer

## Abstract

**Background:**

In recent years, Genome-Wide Association Studies (GWAS) has identified risk variants related to complex diseases, but most genetic variants have less impact on phenotypes. To solve the above problems, methods that can use variants with low genetic effects, such as genetic risk score (GRS), have been developed to predict disease risk.

**Methods:**

As the GRS model with the most incredible prediction power for complex diseases has not been determined, our study used simulation data and prostate cancer data to explore the disease prediction power of three GRS models, including the simple count genetic risk score (SC-GRS), the direct logistic regression genetic risk score (DL-GRS), and the explained variance weighted GRS based on directed logistic regression (EVDL-GRS).

**Results and Conclusions:**

We used 26 SNPs to establish GRS models to predict the risk of biochemical recurrence (BCR) after radical prostatectomy. Combining clinical variables such as age at diagnosis, body mass index, prostate-specific antigen, Gleason score, pathologic T stage, and surgical margin and GRS models has better predictive power for BCR. The results of simulation data (statistical power = 0.707) and prostate cancer data (area under curve = 0.8462) show that DL-GRS has the best prediction performance. The rs455192 was the most relevant locus for BCR (*p* = 2.496 × 10^–6^) in our study.

**Supplementary Information:**

The online version contains supplementary material available at 10.1186/s12894-024-01524-6.

## Introduction

In recent years, Genome-Wide Association Studies (GWAS) have identified risk genetic factors associated with complex diseases. However, in terms of genetic analysis, for large-scale data, single nucleotide polymorphism (SNP) is still challenging for clinical application of complex diseases. At the same time, as a single SNP has little impact on the phenotypes of complex diseases [1], methods that can better use little impact genetic variation, such as the Genetic Risk Score (GRS), have been developed to integrate the risk alleles of selected SNPs into the overall risk score to predict complex diseases [2–4].

The published GRS models, simple count GRS (SC-GRS), direct logistic regression GRS (DL-GRS), and explained variance weighted GRS based on directed logistic regression (EVDL-GRS) have been widely used to evaluate the association between genetic factors and complex diseases [5]. The advantage of the simple count GRS is that the calculation is simple and easy to understand and is most suitable when the effect of SNPs cannot be stably estimated. As this method assumes that all SNPs have the same impact on the disease, which is almost impossible in reality, it is rarely used in the establishment of prediction models On the other hand, DL-GRS assumes that SNPs with greater odds ratio have a greater impact on diseases, thus, compared with the simple count GRS, the hypothesis of DL-GRS is more reasonable, and it is most suitable when the odds ratio of SNPs cannot be accurately estimated through external research. Its disadvantage is that its power to predict the external population could be better. The method assumes that both the odds ratio and the minimum allele frequency (MAF) are the critical factors affecting the disease; however, as it relies on existing data, it also has the disadvantage of poor power in predicting the external population. As each of the three GRSs has its own advantages and disadvantages, the model with the most incredible prediction power is still unknown.

There were several past GWAS related to prostate cancer [6–12]. Approximately 30% of patients with prostate cancer have experienced biochemical recurrence (BCR) within ten years after resection [13], and the critical predictors of BCR include prostate-specific antigen (PSA), the Gleason score, and the pathological stage. However, some studies have found that the prediction accuracy of these factors is limited by the following factors: false positive results may be obtained in screening with PSA, which may lead to overdiagnosis and over-treatment [14]. Even if the Gleason score of different patients is the same, the clinical prognosis will be significantly different [15], as the pathological stage cannot provide long-term information regarding BCR [16]. Due to the limitations of the above predictors and because few studies have explored the use of genetic markers to predict postoperative BCR in Asian populations [17], this study mainly analyzed the genes or loci associated with the BCR of patients with prostate cancer, to reduce the risk of BCR. In addition, the prostate-GRS models have rarely been developed.

As the GRS model with the most incredible prediction power for complex diseases has not been determined, our study used simulation data and prostate cancer data to explore disease prediction power of three GRS models. This study hypothesized that different GRS models may have different discrimination accuracy in predicting the risk of BCR. In addition, clinical gene models that include clinical variables and GRSs may be the better tool to predict BCR; therefore, this study used simulation and real data for hypothesis verification. The results of simulation data and prostate cancer data show that logistic regression GRS has the best prediction power.

## Methods

### Genetic risk score models

As a single SNP has little effect on the phenotype of complex diseases, GRS is used to integrate the risk alleles of the selected SNPs into the overall risk score to predict complex diseases. The following three GRSs were used in this study to assess the association between genetics and complex diseases:

#### Simple count GRS (SC-GRS)

Assume that a SNP is a base pair C and variant T, and C is a risk allele; because it does not have a risk allele, genotype TT is marked as 0; genotypes CT or TC are marked as 1, as they have risk alleles; genotype CC is marked as 2. The $\text{SC}-\text{GRS}=\sum_{i=1}^{k}{SNP}_{i}$, where k is the number of SNPs and ${SNP}_{i}$ is the number of risk alleles. This method is the simplest, as it assumes that all SNPs have the same effect on the disease. Thus, only the numbers of SNPs with risk alleles are calculated [1, 5, 18].

#### Logistic regression GRS (DL-GRS)

The $\text{DL}-\text{GRS}=\sum_{i=1}^{k}{{\beta }_{i}SNP}_{i}$, where k is the number of SNPs, ${SNP}_{i}$ is the number of risk alleles, and ${\beta }_{i}$ is the coefficient of logistic regression. In contrast to SC-GRS, this method considers that SNPs have varied impacts on the disease. Thus, the logistic regression coefficient is taken as the weight and put into the model for calculation [1, 5, 19].

#### Explained variance weighted GRS based on logistic regression (EVDL-GRS)

The $\text{EVDL}-\text{GRS}=\sum_{i=1}^{k}{{W}_{{E}_{i}}SNP}_{i}$, where ${W}_{{E}_{i}}={\beta }_{i}\sqrt{2{MAF}_{i}(1-{MAF}_{i})}$, k is the number of SNPs, ${SNP}_{i}$ is the number of risk alleles, ${\beta }_{i}$ is the coefficient of logistic regression and ${MAF}_{i}$ is the MAF of the *i*^th^ SNP. The risk of SNPs and the MAF are both critical factors for diseases; therefore, this method includes both factors in the model [1, 5, 20].

Single nucleotide polymorphisms (SNPs) are critical factors in assessing disease risk due to their influence on genetic variability and disease susceptibility. The significance of SNPs is often characterized by their odds ratios (OR) and minor allele frequencies (MAF). Studies have shown that SNPs can significantly impact disease risk, with OR measuring the strength of association between a particular SNP and disease occurrence. For example, Barreiro LB et al. (2008) demonstrated that natural selection has driven population differentiation in modern humans through SNPs, highlighting their role in disease susceptibility [21]. Additionally, research on diabetes risk among Middle Eastern populations emphasized the importance of SNPs' OR and MAF in understanding genetic predispositions to the disease [22]​. Further, a study on idiopathic pulmonary fibrosis (IPF) in a Mexican cohort illustrated how specific SNP-SNP interactions can alter disease risk, underscoring the complex nature of genetic influences as assessed by OR and MAF [23]​. Collectively, these studies underscore the importance of SNPs in genetic research, particularly in their capacity to elucidate the genetic underpinnings of disease risk.

Therefore, in this study, we utilized the SC-GRS, which assumes equal effect sizes for all SNPs, followed by the DL-GRS, which incorporates odds ratios. Lastly, we employed the EVDL-GRS, which takes into account both odds ratios and minor allele frequencies.

### Simulation design

This study used SeqSIMLA (version: 2.9.1) [24] to generate simulation data, a simulation sequence and phenotype tool. The reference sequence files provided on the website were generated through simulation and parameter settings as based on the Asian population in the 1000 Genomes Project.

Simulation parameter settings for prevalence, odds ratio, and case–control ratio were meticulously selected to enhance the robustness of our study. The rationale behind these settings is detailed as follows:

#### Prevalence

Prevalence is the proportion of the total population with a particular disease at a specific time. This study aims to explore the predictive ability of genetic risk scores for complex diseases. Therefore, we set the prevalence at 11%, 20%, and 30%, following the guidelines of Che and Motsinger-Reif (2012) [25].

#### Odds Ratio

According to previous simulation studies [26], the minor allele frequency (MAF) of single nucleotide polymorphisms (SNPs) varies with different odds ratios. If the odds ratio is set at 3, the MAF of SNPs will be less than 0.005; for an odds ratio of 2, the MAF will be less than 0.01; for an odds ratio of 1.5, the MAF ranges between 0.01 and 0.05; and for an odds ratio of 1.2, the MAF ranges between 0.05 and 0.1. SNPs with a MAF less than 0.01 are considered rare variants. Given that both common and rare variants can influence disease risk, we set the simulated odds ratios {1, 1.2, 1.5, 1.7, 2, 2.4} to investigate the contributions of both common and rare variants to complex diseases.

#### Case–Control Ratio

The ratio of cases to controls is critical to study design. Commonly used ratios are 1:2, 1:3, or 1:4, which enhance statistical power. Although statistical power increases with a larger ratio, it plateaus when the ratio exceeds 4 [27]. For this study, we utilize prostate cancer data and simulate two datasets: one with a case–control ratio of 1:1, reflecting the actual prostate cancer data, and another with a ratio of 1:4 to achieve enhanced statistical power. Therefore, we set the simulated case–control ratios to 1:1 and 1:4.

The genetic data of 36 different disease models were generated by combining the above three parameter factors, and 1000 simulation datasets were generated by each model. Under the additive genetic model, the association between the selected SNPs and the risk of complex diseases was evaluated by logistic regression analysis, and the odds ratios (OR) of SNPs were analyzed. We compared the GRS of the case group and that of the control group to identify significant differences by Wilcoxon rank-sum test.

### Ethics approval and consent to participate

The cross-sectional study method was used to compare the discrimination between different GRSs. The subjects were patients diagnosed with prostate cancer and underwent radical prostatectomy from 1995 to 2009. The genotype data were sequenced by Axiom™ Genome-Wide CHB 1 Array Plate. Patients with prostate cancer were enrolled in the study, and the PSA was used to determine whether there was BCR. Patients in the case group were defined as patients with prostate cancer and BCR; patients in the control group were defined as patients with prostate cancer but without BCR, and those with genotype or missing questionnaire data were excluded. This study was approved by the institutional review board of Kaohsiung Medical University Hospital (KMU HIRB-2013132). All participants signed written consent forms before conducting the questionnaire survey and sample collection. The primary data of the subjects and the important predictors of BCR were collected by questionnaire. All patients provided written informed consent prior to the study enrolment. The genetic data has been de-identification measures to address potential privacy concerns related to genetic data handling, ensuring that privacy issues are adequately resolved. Additionally, the data will be used exclusively for this study, and upon its completion, the data will be destroyed to ensure its security. The patient inclusion and exclusion criteria have been described previously [28]. Detailed clinicopathological information was obtained from the patient's medical records.

### Data analysis

The 189 patients with prostate cancer who underwent radical prostatectomy were collected from 1995 to 2009. In terms of genotype data, 14,269,821 SNPs were obtained after gene sequencing, and then, after deleting the subjects without genotype data and excluding the SNPs that did not meet the quality control threshold (i.e., minor allele frequency < 0.01, genotype call rates < 0.95, and departure from Hardy–Weinberg equilibrium (i.e., *p*-value < 10 − 4)), 185 subjects and 7,283,541 SNPs remained.

**Fig. 1:**
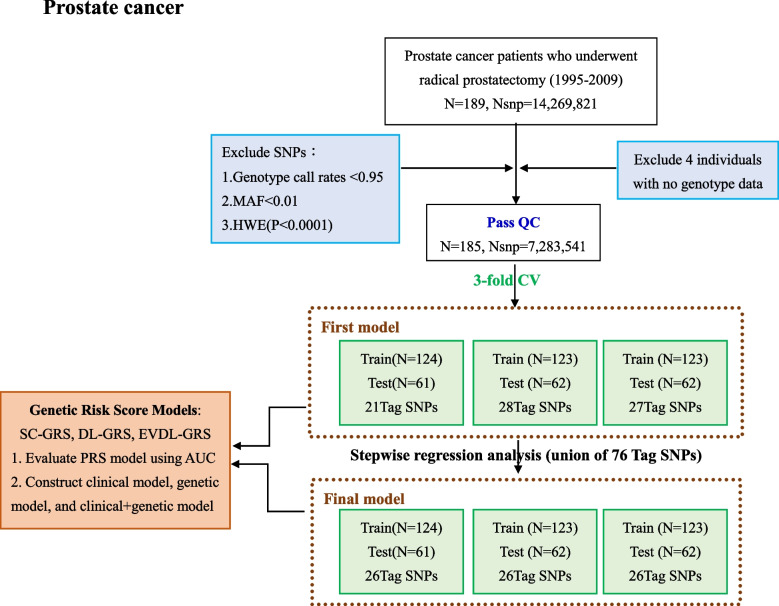
Flowchart of the prostate cancer analyses performed

Prostate cancer databases were analyzed primarily by establishing two genetic models. The detailed process is described below and illustrated in the flowchart (Fig. 1).

#### First Model

The first model used threefold cross-validation (threefold CV) to divide the data into training and testing sets randomly. An additive logistic regression model was employed to analyze the SNPs with BCR as the phenotype, and Tag SNPs with *P* < 0.0001 were selected for subsequent analysis.

#### Second Model (Final Model)

In the second model, SNPs were selected from the Tag SNPs identified in the threefold CV by using a joint set. The SNPs with the most significant impact were retained through stepwise regression analysis and included in the training and testing sets of the threefold CV to form the final model. Under the additive genetic model, the associations between the selected SNPs and the risk of BCR were evaluated using logistic regression analysis, and the odds ratios of the SNPs were determined. A total of 76 SNPs (21 SNPs, 28 SNPs, and 27 SNPs) were selected from the Tag SNPs in the threefold CV using the joint set approach, and SNPs with *P* < 0.05 were retained through stepwise regression analysis.

To investigate the BCR status of prostate cancer patients after radical prostatectomy, the GRSs were divided into four groups (Q1: < 25% percentile, Q2: 25–50% percentile, Q3: 50–75% percentile, Q4: ≥ 75% percentile). The various variables, such as prostate-specific antigen, Gleason score, pathological stage, and PSA were adjusted into the Cox proportional-hazards model as a clinical model. The other model added clinical variables and gene variables, namely, GRS, as the clinical gene model, and the hazard ratios of the two Cox proportional-hazards models were compared. The age at diagnosis, body mass index, months without BCR after surgery, prostate-specific antigen, and the Gleason score of the subjects were analyzed. Independent t-testing was used for continuous variables conforming to a normal distribution, while the Wilcoxon rank-sum test was used for continuous variables with not doing to normal distribution. The continuous variables are presented as mean ± standard deviation, interquartile range (IQR), and range. The chi-square test was used for category variables, which are presented as both number and percentage.

We employed sensitivity analyses to evaluate the robustness of the GRS models across various patient subgroups. Specifically, we conducted three different sensitivity analyses within the context of prostate cancer analysis:

1. Excluding cases with PSA levels below the first quartile (Q1).
2. Excluding cases with PSA levels above the third quartile (Q3).
3. Randomly excluding 25% of the cases.

All data were analyzed using SAS 9.4 (SAS Inc.; Cary, NC, USA) and Plink 2.0 [29].

## Results

### Simulation data

We generated a total of 36 different disease models, and each model generated 1000 simulation data (Supplementary Table S1). After quality control, 10 SNPs with *P* < 0.05 were selected and put into SC-GRS, DL-GRS, and EVDL-GRS to calculate the power of testing and Type I Errors.

**Fig. 2:**
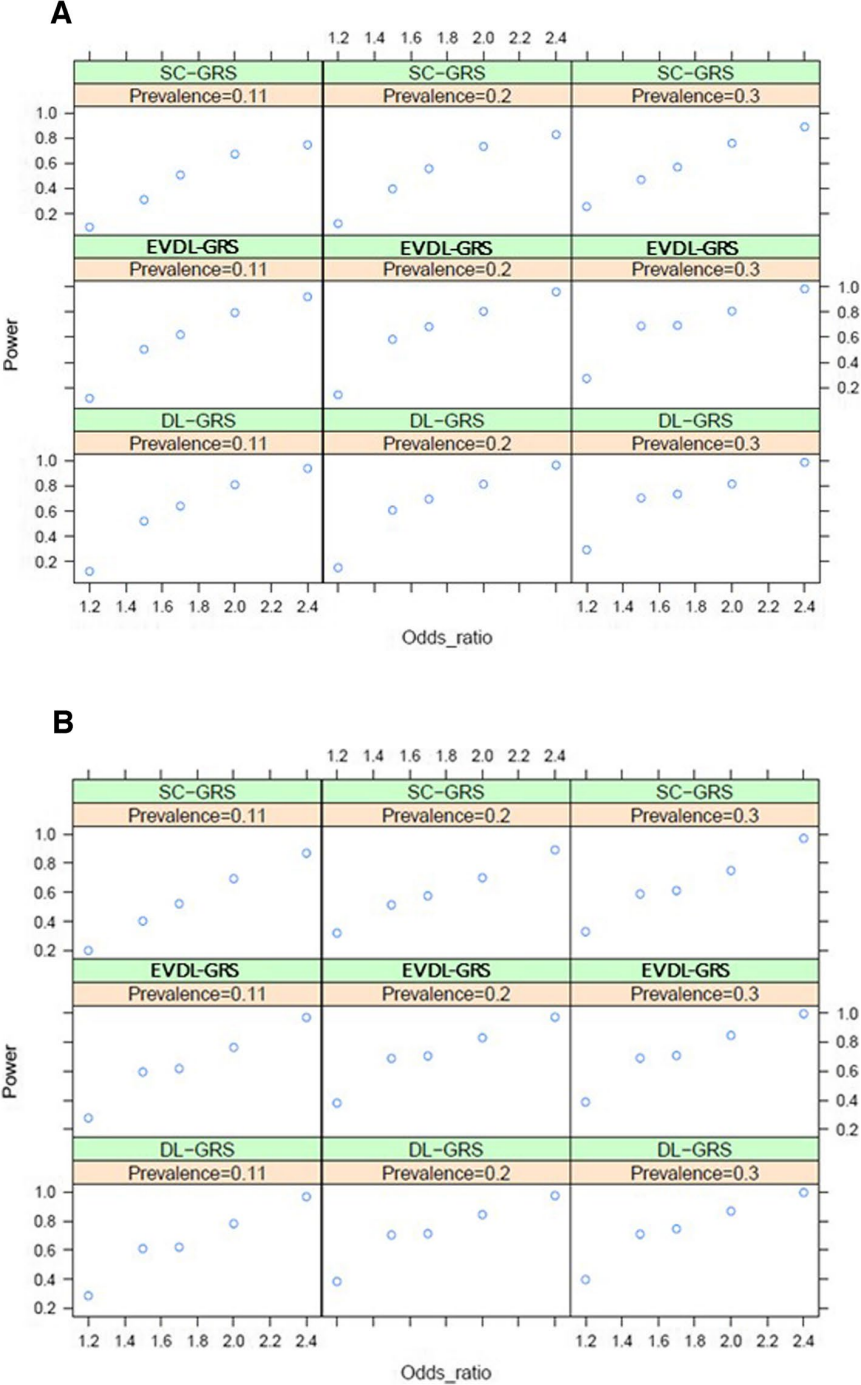
Results of the power in our simulation study under (**A**) case:control = 1000:4000, and (**B**) case:control = 2000:2500

When the prevalence is 0.11, the DL-GRS has the most incredible statistical power of (Power = 0.606), followed by EVDL-GRS (Power = 0.591), while the SC-GRS (Power = 0.467) has the lowest statistical power. When the prevalence is 0.2, the DL-GRS has the most significant statistical power (Power = 0.647), followed by EVDL-GRS (Power = 0.635), while the SC-GRS (Power = 0.528) has the lowest statistical power. When the prevalence is 0.3, the DL-GRS has the most significant statistical power (Power = 0.707), followed by EVDL-GRS (Power = 0.690), while the SC-GRS (Power = 0.589) has the lowest statistical power. In the case of a fixed odds ratio, the statistical powers of SC-GRS, DL-GRS, and EVDL-GRS will increase with the rise of prevalence. In addition, when the prevalence is fixed, the statistical power of all of the 3 GRS models will increase with the odds ratio (Fig. 2 (A)). In either case, compared with the case group = 1000 and control group = 4000, if the case group = 2000 and the control group = 2500, when all the SNPs are put into the SC-GRS, DL-GRS, and EVDL-GRS for calculation, the statistical power is higher (Fig. 2 (A) and Fig. 2 (B)).

The Type I Error of SC-GRS (Type I Error = 0.043) is best controlled, followed by that of EVDL-GRS (Type I Error = 0.055), while the Type I Error of DL-GRS (Type I Error = 0.057) is the highest. Under the condition of a fixed odds ratio, the Type I Error of SC-GRS, DL-GRS, and EVDL-GRS will all increase with the rise of prevalence; when the prevalence is fixed, the Type I Error of DL-GRS is the highest, followed by EVDL-GRS and SC-GRS (Supplementary Table S2). Compared with the case group = 1000 and control group = 4000, if the case group = 2000 and the control group = 2500, when all the SNPs are put into the SC-GRS, DL-GRS, and EVDL-GRS for calculation, the Type I Error is higher (Supplementary Table S2).

### Prostate *cancer*

Table 1 describes the basic demographic and clinical variables of prostate cancer patients, with BCR as the phenotype. Among the 185 patients with prostate cancer, 95 (51.35%) had no BCR, and 90 (48.65%) had BCR. For patients with BCR, the PSA (*p* < 0.001), Gleason score (*p* = 0.043), severity of the pathological stage (*p* = 0.001), and number of patients with positive surgical margin (*p* = 0.004) were all greater than those of patients without BCR. In comparison, the number of months without BCR (*p* < 0.001) was lower than that of patients without BCR, and there was significant difference. There were no significant differences in age at diagnosis, height, weight, body mass index, or lymph node involvement (*p* > 0.05) (Table 1).

**Table 1:** Clinicopathologic characteristics of the study population

Variables	All (*n* = 185)	BCR		*P*-value
		**No (*n* = 95)**	**Yes (*n* = 90)**	
**Age at diagnosis, yr**	65.47 ± 6.36 (Range = 34, IQR = 9)	64.83 ± 6.60 (Range = 34, IQR = 9)	66.14 ± 6.06 (Range = 25, IQR = 9)	0.159
**height, cm**	166.26 ± 5.84 (Range = 30.12, IQR = 8.17)	166.70 ± 5.59 (Range = 24.92, IQR = 8.17)	165.80 ± 6.09 (Range = 30.12, IQR = 9.61)	0.32
**weight, kg**	67.72 ± 8.31 (Range = 43.11, IQR = 11)	68.29 ± 8.09 (Range = 39.40, IQR = 10)	67.11 ± 8.53 (Range = 38.11, IQR = 12.6)	0.335
**BMI (kg/m**^**2**^**)**	24.50 ± 2.78 (Range = 13.14, IQR = 3.45)	24.58 ± 2.67 (Range = 12.26, IQR = 3.69)	24.41 ± 2.90 (Range = 13.14, IQR = 3.48)	0.681
**PSA at diagnosis, ng/ml**	17.65 ± 17.50 (Range = 98.43, IQR = 14.45)	13.63 ± 14.90 (Range = 96.23, IQR = 8.35)	21.89 ± 19.06 (Range = 96.84, IQR = 20.44)	< 0.001
**Gleason score**				
**2–7**	158 (85.41)	86 (90.53)	72 (80.00)	
**8–10**	27 (14.59)	9 (9.47)	18 (20.00)	0.043
**Pathologic T stage**				
**T1-2**	132 (71.35)	78 (82.11)	54 (60.00)	
** > T3**	53 (25.65)	17 (17.89)	36 (40.00)	0.001
**Surgical margin**				
**Negative**	108 (58.38)	65 (68.42)	43 (47.78)	
**Positive**	77 (41.62)	30 (31.58)	47 (52.22)	0.004
**Lymph node involvement**				
**Negative**	181 (97.84)	95 (100.00)	86 (95.56)	
**Positive**	4 (2.16)	0 (0.00)	4 (4.44)	0.054

### 3-fold CV

Prostate cancer databases were analyzed mainly by establishing two models. The first used threefold cross-validation (threefold CV) to randomly divide the data into Training Sets and the Testing Set. The additive logistic regression model was used to analyze the SNPs with BCR as the phenotype, and the Tag SNPs were selected from 84, 130, and 67 SNPs with *P* < 0.0001, respectively. Finally, 21, 28, and 27 SNPs were included in the Training Set and Testing Set for subsequent analysis (Supplementary Table S3). Detailed information, including effect sizes for the 21, 28, and 27 SNPs, is shown in Supplementary Table S3. In the model building, 76 SNPs (21 + 28 + 27 SNPs) were selected from Tag SNPs in threefold CV by way of joint set, and the SNPs with most significant impact were retained by stepwise regression analysis and put into the Training Set and Testing Set of threefold CV as the final model. A total of 26 Tag SNPs was included in the final model for subsequent analysis.

Three analysis results were obtained by threefold CV analysis: 21 to 28 SNPs were selected each time (Supplementary Table S3) for SC-GRS, DL-GRS, and EVDL-GRS, and then, grouped by quartile (Q1, Q2, Q3, Q4), and the differences between the four groups were observed. Regarding the no-BCR proportion of the three GRSs among the four groups in the threefold CV, the proportions of all three GRSs in Q1 were 100% in the Training Set; the higher the score, the more patients with BCR, and the more they are prone to recurrence. This trend was consistent in the Testing Set (Supplementary Table S4).

The Supplementary Table S5 shows the AUC ranges of the Training Sets, as obtained by predicting BCR through SC-GRS, DL-GRS, and EVDL-GRS, which are 0.9878–0.9968, 0.9919–0.9979, and 0.9909–0.9966, respectively; the AUC ranges of the Testing Set are 0.6975–0.7592, 0.7672–0.8462, and 0.7672–0.8495, respectively. The AUC range of the Training Set that explored the clinical variables, such as age at diagnosis, PSA, Gleason score, pathologic T stage, surgical margin, BMI, and lymph node involvement, is 0.7172–0.7710, while that of the Testing Set is 0.7892–0.8244. The AUC ranges of the Training Set that explored both SC-GRS, DL-GRS, and EVDL-GRS and clinic variables are 0.9919–0.9989, 0.9956–1.0000, 0.9951–0.9989, while the AUC ranges of the Testing Set are 0.8366–0.8739, 0.8955–0.9163, and 0.8998–0.9241, respectively. Moreover, the calibration plots of the training and testing sets by threefold CV analysis for predicting BCR under different models are shown in the Supplementary Table S10.

The multivariate Cox proportional hazard model includes clinical variables, such as age at diagnosis, body mass index, PSA, Gleason score, pathologic T stage, and surgical margin. After adjustment of the clinic variables, the results show significant factors in SC-GRS, DL-GRS, and EVDL-GRS (*p* < 0.05) (Supplementary Table S6).

### Final model

After the tag SNPs in the threefold CV were selected using joint sets, the most significant SNPs were retained by stepwise regression analysis and put into the Training Set and Testing Set of the threefold CV as the final model (Fig. 1).

Regarding the 26 SNPs with the most significant influence on BCR, the odds ratio of SNPs to BCR was 0.2748 (95% C.I.: 0.1580–0.4675) to 3.321 times (95% C.I.: 1.7560–6.2830). The rs455192 was the most relevant locus for BCR (*p* = 2.496 × 10^–6^) (Table 2). When 26 SNPs were included in the Training Set, the no-BCR proportion of three GRS in Q1 was 100%; the higher the score, the more patients with BCR, and the more likely they were to recur, and this trend was consistent in the Testing Set (Supplementary Table S7).

**Table 2:** The final genetic model by logistic regression analysis

SNP	chromosome	Gene	GRCh37	Risk allele	OR (95%C.I.)	*P*
rs10893363	11	*PKNOX2* [30]	125,165,779	A	0.4420(0.2877,0.6791)	1.94E-04
rs10910023	1		2,791,143	T	2.1340(1.3720,3.3190)	7.73E-04
rs1126232	5		2,565,001	A	0.3414(0.2124,0.5488)	9.11E-06
rs114309234	3		79,820,723	T	2.0160(1.3010,3.1240)	1.71E-03
rs1157173	4		136,424,005	A	0.4077(0.2587,0.6424)	1.10E-04
rs12055887	7	*GTF2IRD1* [31]	73,989,385	G	0.3950(0.2385,0.6541)	3.06E-04
rs12205723	6	*PLEKHG1* [32]	150,996,756	G	2.2130(1.4060,3.4830)	5.94E-04
rs12615937	2		180,276,164	G	0.2878(0.1496,0.5539)	1.93E-04
rs1325340	13	*DACH1* [33]	72,439,407	T	1.9670(1.2850,3.0100)	1.84E-03
rs146162203	2		95,705,097	C	1.7140(1.1220,2.6180)	1.27E-02
rs16872714	5		4,048,841	C	2.8520(1.7630,4.6140)	1.95E-05
rs17110462	1	*SSBP3* [34]	54,821,382	C	0.4510(0.2719,0.7483)	2.05E-03
rs1972538	14	*COQ6* [35]	74,422,843	C	0.4441(0.2939,0.6711)	1.16E-04
rs2012791	20	*PLCB4* [36]	9,134,107	T	2.1650(1.2890,3.6370)	3.51E-03
rs2221490	5		40,641,763	C	2.3270(1.4950,3.6210)	1.81E-04
rs28641058	1		232,424,623	T	0.5698(0.3638,0.8924)	1.40E-02
rs34160974	9	*MAMDC2* [37]*, MAMDC2-AS1* [38]	72,781,196	C	0.4070(0.2452,0.6757)	5.10E-04
rs34195741	3		147,351,456	C	0.5340(0.3482,0.8191)	4.05E-03
rs4074645	3	*MAP3K13* [39]	185,110,306	G	3.3210(1.7560,6.2830)	2.24E-04
rs455192	17		9,670,373	C	0.2718(0.1580,0.4675)	2.50E-06
rs4850564	2	*LOC105376755*	195,780,804	T	2.6420(1.4000,4.9860)	2.72E-03
rs60256305	20		46,542,553	T	0.3871(0.2210,0.6781)	9.06E-04
rs72642748	13		91,265,212	C	2.3420(1.4790,3.7100)	2.87E-04
rs72918154	18	*ZBTB7C* [40]	45,563,418	C	2.5670(1.5540,4.2380)	2.30E-04
rs76446227	6	*SMOC2* [41]	169,054,760	G	1.8330(1.1750,2.8600)	7.61E-03
rs7985583	13		112,618,602	A	2.5470(1.4790,4.3870)	7.49E-04

The AUC range of the Training Set that explored the clinical variables, such as age at diagnosis, PSA, Gleason score, pathologic T stage, surgical margin, BMI, and lymph node involvement, is 0.7020–0.7592, while that of the Testing Set is 0.7836–0.8000 (Table 3). Table 3 shows the AUC ranges of the Training Sets, as obtained by predicting BCR through SC-GRS, DL-GRS, and EVDL-GRS in the final genetic model (26 SNPs), which are 0.9979–0.9980, 0.9976–0.9989, and 0.9977–0.9992, respectively; the AUC ranges of the Testing Set are 0.9860–0.9979, 0.9999–0.9999, and 0.9958–0.9999, respectively. The AUC ranges of the Training and Testing Set that explored SC-GRS, DL-GRS, and EVDL-GRS and clinic variables are all 0.9999.

**Table 3:** The AUC ranges of the training and testing sets by 3-folds CV analysis for predicting BCR under clinical, genetic, and clinical + genetic models in the final model

	**Prediction Model**^a^	**Genetic Risk Score Models**	**Training set (AUC)**	**Testing set (AUC)**
**First-fold (26 SNP)**	Clinical model	SC-GRS	0.7592	0.7924
	Genetic model		0.9979	0.9860
	Clinical + Genetic model		0.9999	0.9999
	Clinical model	DL-GRS	0.7592	0.7924
	Genetic model		0.9980	0.9999
	Clinical + Genetic model		0.9999	0.9999
	Clinical model	EV_DL-GRS	0.7592	0.7924
	Genetic model		0.9977	0.9999
	Clinical + Genetic model		0.9999	0.9999
**Second-fold (26 SNP)**	Clinical model	SC-GRS	0.7395	0.8
	Genetic model		0.9980	0.9958
	Clinical + Genetic model		0.9999	0.9999
	Clinical model	DL-GRS	0.7395	0.8
	Genetic model		0.9989	0.9979
	Clinical + Genetic model		0.9999	0.9999
	Clinical model	EV_DL-GRS	0.7395	0.8
	Genetic model		0.9992	0.9958
	Clinical + Genetic model		0.9999	0.9999
**Third-fold (26 SNP)**	Clinical model	SC-GRS	0.702	0.7836
	Genetic model		0.9981	0.9979
	Clinical + Genetic model		0.9999	0.9999
	Clinical model	DL-GRS	0.702	0.7836
	Genetic model		0.9976	0.9999
	Clinical + Genetic model		0.9999	0.9999
	Clinical model	EV_DL-GRS	0.702	0.7836
	Genetic model		0.9984	0.9999
	Clinical + Genetic model		0.9999	0.9999

^a^Clinical model: the prediction model with clinical variables including age at diagnosis, PSA, Gleason score, pathologic T stage, surgical margin, BMI, and lymph node involvement; Genetic model: the prediction model with genetic variables according to Genetic Risk Score Models; Clinical + Genetic model: the prediction model with both clinical and genetic variables

Supplementary Table S8 constructs four multivariate Cox proportional hazard models for the Training Set and Testing Set of the final model. These four models contain and use clinical variables, such as age at diagnosis, body mass index, PSA, Gleason score, pathologic T stage, and surgical margin. After adjustment of the clinic variables, the results show that SC-GRS, DL-GRS, and EVDL-GRS were significant factors in both the Training Set and Testing Set (*p* < 0.05).

In the sensitivity analyses, the results indicated that the best outcomes were observed when 25% of the cases were randomly excluded. In contrast, excluding cases with PSA levels below Q1 resulted in the least favorable outcomes, with the AUC decreasing from 0.9976 to 0.9557(the third CV fold)**.** The sensitivity analyses demonstrate that the GRS models are robust across different patient subgroups. Detailed results are presented in Supplementary Table S9.

## Discussion

This study used simulation data and prostate cancer data to compare the power of SC-GRS, DL-GRS, and EVDL-GRS to predict complex diseases, and took the statistical power or AUC, whichever was the higher, as the optimal risk prediction model.

The results of simulation data and prostate cancer data found that, compared with SC-GRS and EVDL-GRS, the best model for predicting BCR in patients with prostate cancer is DL-GRS. GRS has been considered a standard method to evaluate the association between genetics and complex diseases and solve the weak genetic effect of SNP on phenotypes.

Our simulation data show that the higher the prevalence, the greater the statistical power; when the odds ratio is higher, the statistical power also has an upward trend. Compared with the case group = 2000 and the control group = 2500, the statistical power is lower when the case group = 1000 and the control group = 4000, and the three GRS have the same results, among which the statistical power of DL-GRS is the greatest. The higher the prevalence, the higher the Type I Error; compared with the case group = 2000 and the control group = 2500, the Type I Error is lower when the case group = 1000 and the control group = 4000, and the three GRS have the same results, among which the Type I Error of DL-GRS is the greatest. Apart from prevalence, odds ratio, and case: control ratio, the variables affecting the results of the three GRS also include the MAF, as shown in Supplementary Figure S1. This study selected the best model from 36 disease models for further supplementary explanation; that is, the control odds ratio is 1.2 or 2.4 when the case group = 2000, the control group = 2500, and prevalence = 0.3. In terms of the association between the MAF and statistical power of the three GRS, regardless of the risk score model, the statistical power increased with the rise of MAF (Supplementary Figure S1).

In the survival analysis part of our prostate cancer analysis, the GRSs were divided into four groups, and the results show that, compared with the other three groups, the group of patients with the highest score was more prone to recurrence after radical prostatectomy, and these results are consistent among the three GRSs. In the part of disease prediction power, the final model combined the SNPs of threefold CV and then, used stepwise regression analysis to select 26 SNPs and put them into the GRS to predict BCR (Supplementary Table S8), thus, the prediction power is better than that of cross-validation analysis (Supplementary Table S6).

Xin J et al. (2018) [1] was the first to evaluate the risk of colorectal cancer using the multiple GRSs model, and the results showed that SC-GRS model was the best method to predict the risk of colorectal cancer in the external population. However, this method assumes that all SNPs have the same impact on complex diseases. Thus, it is not suitable for the construction of a risk assessment model. Through simulation and real data, Xin et al. also proved that DL-GRS is unsuitable for predicting the external population, as its prediction power may be reduced [1]. Gui L et al. (2014) [42] found that when SC-GRS and DL-GRS were included in a model established with the variables of age, gender, and body mass index, the AUC of the models increased by 0.011 and 0.013, respectively, as compared with the model not including GRS, indicating that DL-GRS has greater prediction power than SC-GRS [42], which is consistent with the results of this study.

Although DL-GRS is the best model for predicting BCR, the weight of this method mainly comes from its research, rather than published literature; therefore, when extrapolating other races, it requires further verification. From the perspective of a whole base group association study, the sample size of the prostate cancer database of this study is small. In addition, due to the lack of survival data, it is impossible to explore the survival status of prostate cancer patients suffering BCR after prostate resection.

In our final genetic model analysis using logistic regression (as shown in Table 2), we found that all the genes listed are associated with previously reported prostate cancer genes [30–41, 43]. Specifically, the human Dachshund1 (*DACH1*) gene encodes a DNA-binding protein that resembles those in the winged helix/Forkhead subgroup of the helix-turn-helix family. Furthermore, studies have shown that *DACH1* expression is reduced, and that overexpression of *DACH1* can inhibit the growth of prostate cancer cell lines [43]. Li Z (2023) discovered a novel role for *DACH1* in maintaining genomic stability by regulating DNA repair. In human prostate cancer, reduced *DACH1* levels, often due to gene deletion or promoter DNA methylation, were correlated with poor clinical outcomes [33]. *PLCB4*, which encodes a positive regulator of the phosphatidylinositol-3-kinase signaling pathway, has been implicated in prostate cancer [36].

In the simulation study, SC-GRS demonstrated the lowest Type I error rate (Type I Error = 0.043) but exhibited lower statistical power (Power = 0.467 at 0.11 prevalence, Power = 0.528 at 0.2 prevalence, Power = 0.589 at 0.3 prevalence). DL-GRS achieved the highest statistical power (Power = 0.606 at 0.11 prevalence, Power = 0.647 at 0.2 prevalence, Power = 0.707 at 0.3 prevalence) and showed robustness with increasing prevalence, although it had the highest Type I error rate (Type I Error = 0.057). EVDL-GRS provided a balanced approach with good power (Power = 0.591 at 0.11 prevalence, Power = 0.635 at 0.2 prevalence, Power = 0.690 at 0.3 prevalence) and moderate Type I error (Type I Error = 0.055). For prostate cancer in the first model (Supplementary Table S5), DL-GRS achieved the best AUC (Training AUC = 0.9919–0.9979, Testing AUC = 0.7672–0.8462), followed by EVDL-GRS (Training AUC = 0.9909–0.9966, Testing AUC = 0.7672–0.8495), while SC-GRS had the lowest AUC (Training AUC = 0.9878–0.9968, Testing AUC = 0.6975–0.7592). Overall, SC-GRS, DL-GRS, and EVDL-GRS exhibited similar performance trends in the simulation and the first model for prostate cancer. However, in the prostate cancer final model (Table 3), incorporating sampling data to union SNP information, along with stepwise regression to retain the 26 most significant SNPs (Table 2), improved the statistical power of SC-GRS relative to DL-GRS and EVDL-GRS. In the final model for prostate cancer, SC-GRS (Training AUC = 0.9979–0.9981, Testing AUC = 0.9958–0.9979), DL-GRS (Training AUC = 0.9976–0.9989, Testing AUC = 0.9958–0.9979), and EVDL-GRS (Training AUC = 0.9958–0.9999, Testing AUC = 0.9958–0.9999) all performed equally well. Therefore, if sampling can be used to provide union SNP information from multiple datasets, SC-GRS, DL-GRS, and EVDL-GRS perform equally well. However, if only a single dataset is available, DL-GRS and EVDL-GRS outperform SC-GRS in terms of statistical power.

Several studies consistently suggest that lifestyle factors such as smoking and nutrition (including whole milk/high-fat dairy, fish, meat, poultry, eggs, dairy, dietary fats, cruciferous vegetables, and tomatoes) are associated with prostate cancer recurrence and mortality [44]. Our study did not account for the potential impact of unmeasured confounders related to these factors on prostate cancer outcomes.

In this study, we utilized the SC-GRS, which assumes equal effect sizes for all SNPs; the DL-GRS, which incorporates odds ratios; and the EVDL-GRS, which accounts for both odds ratios and minor allele frequencies. However, our GRS methods did not consider linkage disequilibrium (LD), which limits the generalizability of our findings beyond the East Asian population. Furthermore, using only three GRS models may not capture the full range of potential insights, as other models could provide different perspectives.

We plan to continue collecting more cases in future studies to enhance the representativeness and reliability of our results. We are confident that with an increased sample size, we will be able to provide more robust and widely applicable conclusions. We also plan to compare the results of future studies with the findings of this study to validate further and deepen our understanding. We look forward to making more in-depth contributions to the research on prostate cancer.

Previous studies have shown that if genetic factors are added to the clinical model, it will have better prediction power [45, 46]. Therefore, in addition to using the potential risk factors for BCR, such as Gleason score, pathological stage, and surgical margin, to establish the clinical model, our study included the above risk factors and genetic factors in the clinical gene model and compared it with the clinical model. Our findings show that adding genetic factors can effectively improve the prediction power of the risk model.

### Supplementary Information

Supplementary Material 1.

## Data Availability

Data is provided within the manuscript or supplementary information files.
